# Predictors of serious findings on bi-directional endoscopy in young patients with anemia and GI symptoms

**DOI:** 10.12669/pjms.344.14391

**Published:** 2018

**Authors:** Faisal Aslam, Abdullah bin Khalid, Faraz Siddiqui, Yamna Jadoon

**Affiliations:** 1 Dr. Faisal Aslam, MBBS, FCPS Medicine. Fellowship in GI, Aga Khan University Hospital, Karachi, Pakistan; 2 Abdullah Bin Khalid, MBBS, FCPS Medicine, FCPS Gastroenterology. Lecturer, Assistant Professor, Dow Medical University, Karachi, Pakistan. Aga Khan University Hospital, Karachi, Pakistan; 3 Faraz Siddiqui, Senior Instructor (Research), Department of Medicine, Aga Khan University Hospital, Karachi, Pakistan; 4 Yamna Jadoon, Undergraduate Medical Student, Aga Khan University Hospital, Karachi, Pakistan

**Keywords:** Endoscopy, GI symptoms, Iron deficiency anemia

## Abstract

**Background and Objective:**

Iron deficiency anemia (IDA) has been cited as the most common cause of anemia globally. Gastrointestinal (GI) lesions are amongst the common cause of IDA. Endoscopic evaluation is the most effective way to investigate the IDA. The aim of this study was to show the association of alarming GI symptoms with abnormal endoscopic findings and to cut off the burden and cost of unnecessary endoscopies.

**Methods:**

This is cross sectional study of anemic patient who underwent upper and lower GI endoscopies in Aga Khan University Hospital, Karachi between July-December 2016.

**Results:**

Total 243 patients were identified after excluding ineligible patients. The mean age of subjects was 31.9 ± 6.1 years with a slight over-representation of females (57.4%). 149 (61.31%) patients underwent only upper GI endoscopic evaluation, and 83 (34.15%) patients on whom bi-directional endoscopy was performed (upper and lower). The remaining 11 (4.52%) patients underwent colonoscopy only. 16 (6.6%) subjects had negative findings on evaluation, while gastritis and serious findings were observed in 175 (72.0%) and 52 (21.4%) patients respectively. We found that patients with alarm features such as dysphagia (aOR: 2.07, 95%CI: 0.12-34.1), altered bowel habits (aOR: 1.64, 95%CI: 0.44-6.09) and weight loss (aOR: 1.25 95%CI: 0.54-2.85) demonstrated higher odds of serious findings on endoscopic evaluation as compared to the reference category, however they were not independently associated.

**Conclusion:**

Most of our patients had non-malignant pathologies, while alarm features were not found to be useful predictors of serious findings.

## INTRODUCTION

According to the World Health Organization anemia is defined as a hemoglobin (Hb) level of less than 12.0 g/dl in women and 13.0 g/dl in men.^1^ Iron deficiency anemia (IDA) has been cited as the most common cause of anemia globally.^2^ Endoscopies are an effective way for evaluating anemia in the hospital setting. In a large study among patients with Gastrointestinal (GI) symptoms and anemia, upper (GI) lesions were found in 62% patients comprising predominantly peptic ulcer disease, followed by lower GI lesions (chiefly hemorrhoids).^3^ A study from the Holland found that dyspepsia with anemia was the most common reason for performing endoscopies in young patients. The cause of anemia was picked up in upper GI endoscopies (EGD) in most of these patients. Most patients with serious diagnoses found in gastroscopies had alarming symptoms also. For example, gastric cancer was more frequent in patients with anorexia or weight loss and esophageal cancer in those with dysphagia.^4^ In contrast, in a multicenter based American study, alarming symptoms were not significantly associated with serious findings on endoscopies. 21% of their sample of patients with isolated dyspepsia without alarm symptoms had serious findings on endoscopy. One of the large multicenter study showed that the presence of alarming symptoms as predictors of pathologic endoscopic findings could result in many cases of missed diagnosis.^5^

Asymptomatic patients also present a challenge as they may have disease which can significantly progress by the time. A study from South Asia found that increasing age, low mean corpuscular volume and positive fecal occult blood testing were useful predictors of relevant endoscopy findings in asymptomatic patients with IDA.^6^

Little regional evidence is available about the approach to young patients with IDA who have GI symptoms. Considering that both GI symptoms and anemia are very common it is important to identify which patients genuinely need endoscopies. Our study aims to address this gap in knowledge.

## METHODS

All young patients aged 18-40 years of age with IDA having GI symptoms who underwent upper and/or lower GI endoscopies between July 2016 to December 2016 at Aga Khan University Hospital Karachi were included in the study and patients having active bleeding (per rectal bleed, haematemesis, melena, epistaxis and menorrhagia) were excluded.

Patients data including demographics, comorbid e.g. Diabetes, Chronic kidney disease, hypothyroidism, GI symptoms and physical examination, complete blood count, tissue transglutaminase (TTG), creatinine, vitamin D and iron studies were recorded for all patients.

Following lesions were considered as source of IDA on EGD: Esophagitis, gastric and duodenal ulcers, carcinoma, polyps, gastritis or duodenitis, candidiasis, esophageal and duodenal strictures, portal gastropathy and esophageal varices, gastric and small bowel mass, celiac disease.

Following lesions were considered as source of IDA on colonoscopy: colonic mass, polyps, vascular ectasia, colonic ulcers, colitis, hemorrhoids, ulcers, rectal erythema and strictures.

We categorized the endoscopic findings as normal, abnormal and serious.

Normal: Patient who had normal examination.

Abnormal: Patients who were found to have one or more of the findings e.g gastric erythema (Gastritis), duodenal erythema (Duodenitis), esophageal candidiasis, small varices on EGD, small hemorrhoids, nonspecific colitis or erythema on colonoscopy.

Serious: Patients who were found to have one or more of the findings, e.g, malignancy, large arterio-venous malformations, large GI ulcers (> 2cm), polyps more than 1 cm in size, strictures and celiac disease.

Following variables were investigated for each outcome variable age (years), gender, Hb level (gm/dl), family history of cancer alarming symptoms, TTG levels, vitamin D levels, and presence of co-morbidities including diabetes and chronic kidney disease.

### Statistical Analysis

We calculated the frequency of endoscopic findings for total sample and by type of investigation performed. We also compared the demographic profile, co-morbid, laboratory values, alarm and non-alarm features among our sample. Positive endoscopic findings were summarized by type of examination performed, i.e., EGD or colonoscopy. Mean and standard deviation were used for quantitative variables whereas absolute and relative frequencies were reported for categorical variables. For statistical comparison at bivariate level, t-test and chi-square tests were used. Alternatively, the fisher exact test was used for variables low expected cell counts. A multivariable, binary logistic regression analysis was used to identify factors associated with serious findings among anemic patients undergoing endoscopy. For this analysis, we combined the categories of patients with normal and abnormal findings, which were taken as the reference group. Using a step wise approach, alarming features were entered into the model, followed by variables with p <0.2 at bivariate level. P-values <0.05 were considered statistically significant. Crude and adjusted odds ratios were reported along with 95% confidence intervals for variable retained in the final model. The data entry and analysis was performed on STATA v12.0 (STATA Corp., Inc.)

## RESULTS

We extracted data for 260 adult patients with iron deficiency anemia, aged between 18-40 years who presented for EGD or colonoscopy between July and December, 2016. After excluding ineligible cases, a total sample of 243 patients were obtained. This included 149 (61.31%) patients who underwent only upper GI endoscopic evaluation, and 83 (34.15%) patients on whom bi-directional endoscopy was performed. The remaining 11 (4.52%) patients underwent colonoscopy only.

**Fig.1:**
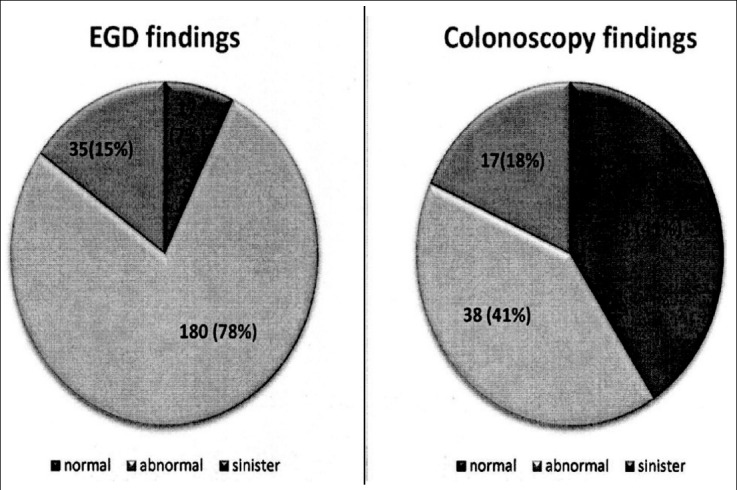
Distribution of abnormal and serious findings by type of examination performed.

Overall, the mean age of subjects was 31.9 ± 6.1 years with a slight over-representation of females (57.4%). There were no differences in age between males and females (31.5±6.05 v 32.2±6.19, p=0.37). In our sample, only 16 (6.6%) subjects had negative findings on evaluation, while gastritis findings and serious findings were observed in 175 (72.0%) and 52 (21.4%) patients, respectively (Fig.1). We found no significant differences with respect to demographic and comorbid profile however, a downward trend in Hb levels was observed in those with abnormal and serious findings, compared to normal (Table-I). In the EGD gastritis (55.5%), varices (16.0%) and ulcers (11.4%) were the most common findings, whereas in colonoscopy, hemorrhoids (8.7%), colitis (4.5%) and colonic ulcers (4.1%) were commonly seen (Table-II).

**Table-I:** Demographic profile, clinical characteristics and symptoms of patients undergoing endoscopy (n=243).

	Normal n (%)	Abnormal n (%)	Serious n (%)	p-value
Total: 243	16 (6.6)	175 (72.0)	52 (21.4)	
*Demographic*				
Age*	31.4 (5.6)	32.0 (6.0)	31.5 (6.7)	0.80
Male	6 (37.5)	69 (39.4)	28 (53.8)	0.16
*Co-morbids*				
Diabetes**	0 (0.0)	21 (12.0)	4 (7.7)	0.25
CKD	11 (4.5)	6 (3.4)	5 (9.6)	0.11
Hypothyroidism	1 (6.25)	10 (5.7)	2 (3.8)	0.85
*Labs*				
Hemoglobin*	8.9 (1.5)	8.4 (2.1)	7.4 (2.4)	0.03
Vitamin D*	14.2 (4.9)	16.5 (13.3)	15.8 (18.3)	0.96
TTGs**	0 (0)	4 (2.3)	6 (11.5)	0.009
*Symptoms*				
Alarm symptoms	2 (12.5)	57 (32.5)	17 (32.7)	0.24
Non-alarm symptoms (upper)	5 (17.8)	59 (32.8)	12 (34.3)	0.26
Non-alarm symptoms (lower)	52 (27.6)	19 (50.0)	5 (29.4)	0.02

* mean (SD), p-value based on one-way analysis of variance

** fisher’s exact test based on expected cell counts <5

**Table-II:** Summary of upper and lower GI findings on endoscopy.

Upper GI findings		Lower GI findings	

	n (%)		n (%)
Gastritis	135(55.5)	Hemorrhoids	21 (8.7)
Varices	39 (16.0)	Colitis	11 (4.5)
Gastric/duodenal ulcer	28 (11.4)	Colon ulcer polyp	10 (4.1)
Candidiasis	4 (1.6)	Colorectal mass	4 (1.6)
Esophageal/duodenal stricture	3 (1.2)	Rectal erythema	3 (1.2)
Gastric/jejunal mass	3 (1.2)	Stricture	2 (0.8)
Polyp	1 (0.4)		1 (0.4)
Angiodysplasia	1 (0.4)		
Duodenitis	1 (0.4)		

Overall, weight loss (20.1%) and altered bowel movements (5.7%) were the most common alarm symptoms, and were found to predominantly occur among patients with positive findings. Most common non-alarm symptoms in upper GI included upper abdominal pain (26.3%), dyspepsia (22.6%) and reflux (3.7%) while in the lower GI it constituted diarrhea (13.6%) and constipation (4.1%). The presence of individual alarm or non-alarm symptoms did not differ among patients with normal, abnormal or serious findings (Table III and IV). We performed a multivariable logistic regression analysis to identify factors associated with serious findings, including alarm symptoms and other potential predictors. In our adjusted model, patients with alarm features such as dysphagia (aOR: 2.07, 95%CI: 0.12-34.1), altered bowel habits (aOR: 1.64, 95%CI: 0.44-6.09) and weight loss (aOR: 1.25 95%CI: 0.54-2.85) demonstrated higher odds of serious findings on endoscopic evaluation as compared to the reference category, however were not independently associated. In contrast, presence of severe anemia (Hb<7gm) (aOR: 4.22, 95%CI: 2.04-10.71) was independently associated with serious findings (Table-IV).

**Table-III:** Crude and Adjusted logistic regression estimates for factors associated with serious findings in anemic patients undergoing endoscopic examination.

Variable	Crude Odds ratio	95%CI	p-value^crude^	Adjusted Odds ratio	95% CI	p-value^adj^
Dysphagia	1.85	0.16-20.84	0.61	2.07	0.12-34.1	0.51
Altered Bowel	1.50	0.45-5.01	0.50	1.64	0.44-6.09	0.45
Weight loss	1.08	0.50-2.29	0.84	1.25	0.54-2.85	0.59
Anemic status*						
Moderate anemia	1.26	0.55-2.88	0.57	1.30	0.54-3.08	0.55
Severe anemia	4.22	1.91-9.30	<0.001	4.69	2.04-10.71	<0.001
Positive TTGs	6.09	1.65-22.5	0.007	6.59	1.59-27.20	0.009

* mild anemia as reference category

**Table-IV:** Detailed distribution of alarm and non-alarm features among patients undergoing endoscopy (n=243).

	Normal n (%)	Abnormal n (%)	Serious n (%)
*Alarming symptoms*			
Weight loss	2 (12.5)	36 (20.5)	11 (21.1)
Altered bowel	0 (0.0)	10 (5.7)	4 (7.7)
Persistent vomiting	0 (0.0)	4 (2.3)	1 (1.9)
Decreased appetite	0 (0.0)	5 (2.8)	0 (0.0)
Dysphagia	0 (0.0)	2 (1.1)	1 (1.9)
*Non-alarming symptoms (upper GI)*			
Dyspepsia	8 (50.0)	37 (21.1)	10 (19.2)
Upper abdominal pain	2 (12.5)	48 (27.4)	14 (26.9)
Reflux	0 (0)	9 (5.14)	0 (0)
*Non-alarming symptoms (upper GI)*			
Diarrhea	2 (12.5)	24 (13.7)	7 (13.4)
Constipation	0 (0)	8 (4.5)	2 (3.8)
Lower abdominal pain	1 (6.2)	0 (0)	0 (0)

## DISCUSSION

Our study is the first to explore associations with the presence of serious endoscopic findings among young patients with IDA and GI symptoms in a Pakistani hospital based setting. We analyzed key alarm features as well as demographic and clinical factors against the presence of serious findings on endoscopies, and found that alarming features do not independently predict major endoscopic findings in the upper or lower GI tract. Among other factors we found severe anemia levels to be positive predictors in our population.

In our study the majority of patients had upper GI involvement, with colonoscopic findings in only one fifth of cases. This is an interesting finding in contrasts with western data in which frequency of lower GI lesions was equal to, or higher than in the upper GI tract.^7^

Alarming symptoms and anemia have routinely been used as predictors of GI malignancies.^8^ Previous guidelines recommend performing endoscopic evaluation in the presence of alarm features regardless of patient’s age.^9^-^11^ Nevertheless, consequent studies of dyspeptic patients have shown alarm features to be of limited significance.^12^,^13^ In our analyses, we found that although alarm features were more common in patients with serious findings, there was no statistically significant association between the two. A possible explanation of low number of malignancies in our sample could be that our sample consisted of young patients in which non-malignant pathologies are usually higher. Some other studies showed higher numbers of malignancies range from 10% to 50%.^14^ Administrative database studies have shown that alarm features in patients younger than 60 years have low positive predictive value and have limited value in deciding if patients should be referred for endoscopy.^15^-^18^ We found patients with severe anemia were significantly more likely to have serious findings. Most of the patients in our sample had gastritis, which is usually caused by H.pylori, a leading cause of IDA.^19^,^20^ Among patients with serious findings, gastric ulcers were the predominant condition.

There is limited data in the Asian population about endoscopic evaluation in IDA patients with gastrointestinal symptoms. Available data is from a very old cohort and shows that the prevalence of endoscopic lesions is up to 70%.^21^-^23^ This is a troubling number and warrants further study in this population.

We have excluded all patients with active GI blood loss as it is the standard of care in our practice to conduct endoscopies with these indications. This highlights the importance of our study as it focuses on evaluating patients who had chronic anemia with no overt blood loss. Our population consisted of young patients aged between 18-40 years, which differentiates our study from other studies which were generally conducted on all age groups. Our study is conducted at single centre and the sample size was lesser in comparison to other studies.

## CONCLUSION

Our study provides first insight into association with serious endoscopic findings in a young symptomatic population with IDA. Most of our patients had non-malignant pathologies, while alarm features were not found to be useful predictors of serious findings. Hence the decision to undertake endoscopy needs to be in the patient’s best interest in terms of efficacy and cost effectiveness. Attention is drawn to the need for less invasive and better alternates to endoscopy. We suggest such patients should be observed and given a medication trial for symptomatic treatment and dyspepsia initially.

### Author`s Contribution

**FA** conceived the study, help in data collection and discussion writing.

**AK** had taken part in finalizing the discussion and methods part.

**FS** had taken part in statistics and results.

**YJ** had role in data collection and discussion writing.
